# Effects of Chronic Dopamine D2R Agonist Treatment and Polysialic Acid Depletion on Dendritic Spine Density and Excitatory Neurotransmission in the mPFC of Adult Rats

**DOI:** 10.1155/2016/1615363

**Published:** 2016-03-23

**Authors:** Esther Castillo-Gómez, Emilio Varea, José Miguel Blasco-Ibáñez, Carlos Crespo, Juan Nacher

**Affiliations:** ^1^Neurobiology Unit, BIOTECMED, Cell Biology Department, Universitat de València, 46100 Burjassot, Spain; ^2^Fundación Investigación Hospital Clínico de Valencia, INCLIVA, 46010 Valencia, Spain; ^3^CIBERSAM, Centro de Investigación Biomédica en Red Salud Mental, 28029 Madrid, Spain

## Abstract

Dopamine D2 receptors (D2R) in the medial prefrontal cortex (mPFC) are key players in the etiology and therapeutics of schizophrenia. The overactivation of these receptors contributes to mPFC dysfunction. Chronic treatment with D2R agonists modifies the expression of molecules implicated in neuronal structural plasticity, synaptic function, and inhibitory neurotransmission, which are also altered in schizophrenia. These changes are dependent on the expression of the polysialylated form of the neural cell adhesion molecule (PSA-NCAM), a plasticity-related molecule, but nothing is known about the effects of D2R and PSA-NCAM on excitatory neurotransmission and the structure of mPFC pyramidal neurons, two additional features affected in schizophrenia. To evaluate these parameters, we have chronically treated adult rats with PPHT (a D2R agonist) after enzymatic removal of PSA with Endo-N. Both treatments decreased spine density in apical dendrites of pyramidal neurons without affecting their inhibitory innervation. Endo-N also reduced the expression of vesicular glutamate transporter-1. These results indicate that D2R and PSA-NCAM are important players in the regulation of the structural plasticity of mPFC excitatory neurons. This is relevant to our understanding of the neurobiological basis of schizophrenia, in which structural alterations of pyramidal neurons and altered expression of D2R and PSA-NCAM have been found.

## 1. Introduction

Impairments of dopaminergic neurotransmission affecting medial prefrontal cortex (mPFC) function are one of the core features of several psychiatric disorders, including schizophrenia. Indeed, a major hypothesis suggests that excessive dopamine D2 receptor (D2R) activation in this area might contribute to prefrontal dysfunction and cognitive deficits in schizophrenia [1, 2]. Moreover, recent evidences indicate that, in addition to these neurochemical imbalances, changes in the plasticity of prefrontocortical circuits may also underlie the etiopathogenesis of this disorder [3]. In this regard, it is important to note that decreased dendritic spine density on mPFC pyramidal neurons is one of the most consistent findings in the brain of schizophrenic patients and animal models of this disorder [4, 5] and after experimental manipulation of mPFC dopamine levels [6].

Structural changes in neurons are likely to be mediated by the expression of cytoskeletal proteins and cell adhesion molecules. One of the molecules that have received more attention recently is the polysialylated form of the neural cell adhesion molecule (PSA-NCAM). NCAM can incorporate long chains of PSA, which give it antiadhesive properties and, consequently, allow neurons to participate in plastic events, such as neuronal migration and the growth or remodeling of neurites, spines, and synapses [7–9]. In adult mammals, this molecule is expressed in cerebral regions where neuronal structural plasticity has been described, such as the mPFC [10], where it is exclusively expressed by interneurons [11]. Interestingly, our laboratory found that PSA-NCAM expressing interneurons in the mPFC of adult rats express D2R and that the manipulation of dopaminergic neurotransmission in this region leads to intense changes in the expression of PSA-NCAM. These changes occur in parallel to changes in the expression of synaptic proteinsand moleculesrelated to inhibitory neurotransmission (synaptophysin and GAD67). The expression of all these molecules is reduced after dopamine depletion (6-hydroxydopamine) or treatment with a D2R antagonist (haloperidol), but it is increased after treatment with a D2R agonist (PPHT) [12]. In a following study, we showed that the presence of PSA on NCAM is necessary for these D2R-induced changes in synapses and inhibitory neurotransmission, since they are blocked when the polysaccharide is ablated from NCAM in the mPFC. Moreover, both PPHT treatment and PSA depletion have a strong impact on the perisomatic inhibitory baskets on mPFC pyramidal neurons, increasing the density of perisomatic puncta expressing GAD65/67 and parvalbumin (PV) [13].

Together, these data strongly suggest the involvement of PSA-NCAM in the plasticity of prefrontocortical inhibitory system, and since interneurons are critical for the regulation and synchronization of pyramidal neurons, these changes in inhibitory circuits should likely also affect these excitatory cells.

In order to better understand the influence of D2R and PSA-NCAM in the structural plasticity of mPFC excitatory circuits, we have replicated our experiment using chronic D2R agonist (PPHT) treatment in rats in which PSA had been previously ablated from the mPFC using the specific enzyme endoneuraminidase-N (Endo-N) [13]. In particular, we have studied whether PPHT affects the degree of excitation in the mPFC (expression of vesicular glutamate transporter-1, VGLUT1) or the structure and connectivity of pyramidal neurons in the mPFC (dendritic spine density and peridendritic GABAergic innervation) and whether these effects could be blocked, potentiated, or reduced by a previous PSA depletion.

## 2. Materials and Methods

### 2.1. Animals and Ethics Statement

Twenty-four male Sprague-Dawley rats (3 months old; Harlan Interfauna Ibérica S.L., Barcelona, Spain) were used in this study (6 rats in each of the four experimental groups). Animals were housed in groups of 3 in a temperature- and humidity-controlled environment and maintained on a 12 h light/dark cycle with food and water available* ad libitum*. Rats were allowed to habituate to our facilities for one week prior to the start of the experiments.

All animal experimentation was conducted in accordance with the Directive 2010/63/EU of the European Parliament and of the Council of 22 September 2010 on the protection of animals used for scientific purposes and was approved by the Committee on Bioethics of Universitat de València. Every effort was made to minimize the number of animals used and their suffering.

### 2.2. Polysialic Acid Depletion and D2R Agonist Chronic Treatment

Rats were deeply anaesthetized (5 mg/g xylazine and 0.5 mL/kg ketamine i.p.) and placed in a stereotaxic instrument (David Kopf Instruments, Tujunga, CA). A 10 *μ*L of FlexiFil taper tip syringe (World Precision Instruments Inc., Sarasota, FL) was then positioned in the secondary motor cortex as described before [13], using the following coordinates from the atlas of Paxinos and Watson [14]: Bregma +1,70 mm, Lateral ±1,00 mm, and Deep −0,80 mm. The needle was left in position for 1 minute and then 1 *μ*L of the enzyme Endo-N (0.7 U/*μ*L in glycerol; AbCys, Paris, France) was injected over 1 minute period into one hemisphere in twelve rats. Control rats (*n* = 12) received the same amount of the vehicle solution (NaCl 0.9% and glycerol, 1 : 1). After the injection was completed, the needle was left in place for 2 minutes to reduce reflux of the solution into the track and then was withdrawn. The Endo-N is a phage enzyme that specifically cleaves alpha-2,8-linked sialic acid polymers with the minimum chain length of 8. It diffuses rapidly throughout the brain and removes all detectable PSA within 1 day for 3-4 weeks [15]. The contralateral hemisphere did not receive any injection because pilot experiments demonstrated that Endo-N also diffuses to contralateral mPFC. After recovery from anesthesia, rats were returned to their cages in the colony room, where they remained for one week prior to the start of pharmacological treatment.

Seven days after the intracranial injection, rats which had received Endo-N (*n* = 12) were randomly separated in 2 groups (*n* = 6) and either selective dopamine D2R agonist 2-(N-Phenethyl-N-propyl) amino-5-hydroxytetralin hydrochloride (PPHT, 1.5 mg/kg in 0.9% NaCl solution; Sigma-Aldrich) or saline (0.9% NaCl solution) was administered to the rats intraperitoneally (i.p.) once daily for 7 consecutive days as described before [12, 13]. The same procedure was followed for rats that had received intracranial vehicle injection (*n* = 12). Animals were perfused transcardially 24 hours after the last i.p. injection.

### 2.3. Histological Procedures

Rats were perfused transcardially under deep chloral hydrate anesthesia, first for 1 minute with NaCl 0.9% and then for 30 minutes with 4% paraformaldehyde in sodium phosphate buffer (PB) 0.1 M, pH 7.4. Thirty minutes after perfusion, brains were extracted from the skull and their hemispheres were separated.

The ipsilateral hemispheres relative to the side of injection were cryoprotected with 30% sucrose in PB 0.1 M (4°C) for 48 hours and then cut in coronal sections (50 *μ*m) with a freezing-sliding microtome (Leica SM2000R; Leica, Nussloch, Germany). Slices were collected in 10 sequential subseries and stored at −20°C in a cryoprotective solution until used (30% glycerol, 30% ethylene glycol in PB 0,1 M).

The contralateral hemispheres were washed in cold PB 0.1 M (4°C) for one day and then cut in 5 mm thick coronal blocks. Two 50 *μ*m sections containing mPFC were obtained from all blocks with a vibratome (Leica VT 1000E) and then the 5-mm thick blocks were impregnated using the Golgi method as described below.

### 2.4. Immunohistochemistry for Conventional Light Microscopy

In order to check whether PSA had been depleted by Endo-N in the ipsilateral and contralateral hemispheres, one 50 *μ*m thick subseries from each hemisphere and each animal was processed by “free-floating” immunohistochemistry using the avidin-biotin-peroxidase (ABC) method as described before [10, 12]. To analyze whether Endo-N injection and/or PPHT treatment induced changes on the neuropil expression of VGLUT1, one 50 *μ*m thick subseries from each animal was processed using the same method.

In brief, sections were first incubated for 1 minute in an antigen unmasking solution (0.01 M citrate buffer, pH 6) at 100°C. After cooling down sections to room temperature, they were incubated with 3% H_2_O_2_ in phosphate buffered saline (PBS) for 10 minutes to block endogenous peroxidase activity. After this, sections were treated for 1 hour with 10% normal donkey serum (NDS; Jackson ImmunoResearch Laboratories, West Grove, PA) in PBS with 0.2% Triton-X100 (Sigma-Aldrich, St. Louis, MO) and then they were incubated overnight at room temperature with mouse IgM anti-PSA-NCAM (1 : 700, AbCys) or guinea pig IgG anti-VGLUT1 (1 : 2000; Millipore) primary antibody in PBS with 0.2% Triton-X-100 and 5% NDS. After washing, sections were incubated for 2 hours at room temperature with donkey anti-mouse IgM, donkey anti-mouse IgG, or donkey anti-guinea pig IgG biotinylated secondary antibody (1 : 400; Jackson ImmunoResearch Laboratories), followed by an avidin-biotin-peroxidase complex (ABC; Vector Laboratories, Peterborough, UK) for 1 hour in PBS. Color development was achieved by incubating with 0.05% 3,3′-diaminobenzidine tetrahydrochloride (DAB; Sigma-Aldrich) and 0.033% H_2_O_2 _for 4 minutes.

Finally, sections were mounted on slides, dried for one day at room temperature, dehydrated with ascending alcohols, and rinsed in xylene. After this, sections were coverslipped using Eukitt mounting medium.

All the studied sections passed through all procedures simultaneously in order to minimize any difference from immunohistochemical staining itself. To avoid any bias in the analysis, all slides were coded prior to analysis and the codes were not broken until the experiment was finished.

**Figure 1:**
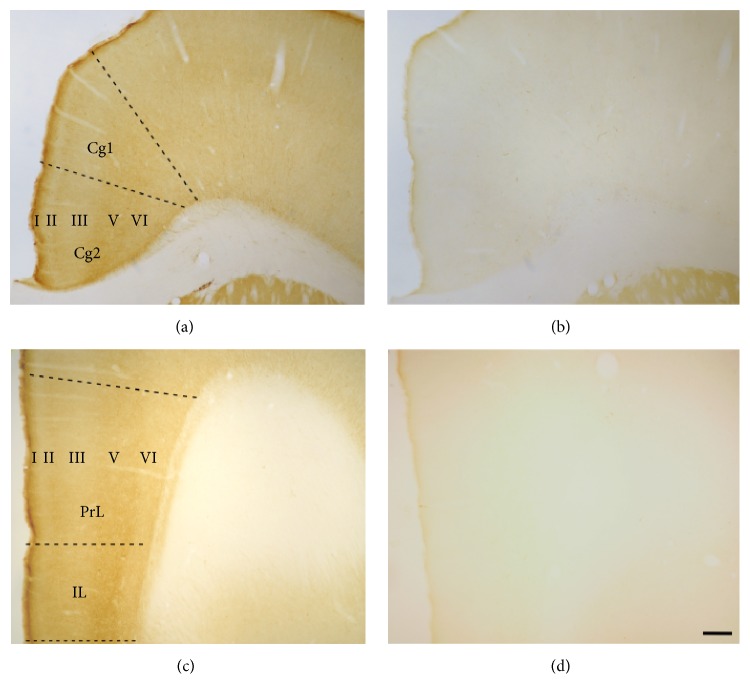
Microphotographs of the rat mPFC cortex showing PSA-NCAM immunostaining in control (a, c) and Endo-N treated rats (b, d). Pictures (a) and (b) show the dorsal (Cg1) and ventral cingulate cortices (Cg2) and pictures (c) and (d) show the infralimbic (IL) and prelimbic (PrL) cortices. Note in (b) the lack of PSA-NCAM expression in Cg1 and Cg2 (as observed in (d) for IL and PrL), but not in the striatum, demonstrating the effectiveness of Endo-N treatment. Roman numbers indicate cortical layers. Scale bar: 100 *μ*m. Stereotaxic coordinates: Bregma +1.80 mm, Interaural 10.80 mm for figures (a) and (b); Bregma +3.00 mm, Interaural +12.00 mm for figures (c) and (d) [14].

PSA-NCAM expression in the mPFC remained undetectable 14 days after Endo-N injection (Figure 1).

### 2.5. Quantification of VGLUT1 Neuropil Immunoreactivity

The intensity of VGLUT1 immunoreactivity in the mPFC neuropil was determined using a previously described methodology [12, 13].

In brief, three sections per animal were selected randomly from the following coordinates infralimbic cortex: Bregma 2.70 to 2.20 mm; prelimbic cortex: Bregma 3.70 to 2.20 mm; cingulate cortex: Bregma 1.70 to −0.26 mm [14]. Sections were examined in an Olympus CX41 microscope under bright-field illumination, homogeneously lighted, and digitalized using a CCD camera. Photographs of the different areas and layers were taken under 20x magnification. A Nissl stain in alternate series of sections was used for determining layer and area boundaries within medial prefrontal cortex regions, based on cytoarchitectural differences across layers and areas. Grey levels were converted to optical densities (OD) using Image J software (NIH). Means were determined for each experimental group and data were subjected to repeated measures ANOVA (followed by Bonferroni's post hoc test for multiple pairwise comparisons) using the IBM SPSS software (version 19).

Region (infralimbic, prelimbic, dorsal cingulate, and ventral cingulate cortices) and layer (I, II, III, V, and VI) were considered as within-subjects variables (repeated measures variables) and treatment (Endo-N/PPHT, Endo-N/Control, Control/PPHT, and Control/Control) was as between-subjects variable. The sphericity assumption in repeated measures variables and the homogeneity of variances in between-subjects variables were first assessed by means of Mauchly's test of sphericity and Levene's test for homogeneity of variances.

### 2.6. Golgi Method and Analysis of Dendritic Spine Density on mPFC Pyramidal Neurons

Five mm thick coronal slices were processed using the Golgi-Colonnier method with some modifications. In brief, slices were postfixed with 3% potassium dichromate and 5% glutaraldehyde for 7 days at 4°C and then impregnated with 0.75% silver nitrate solution for 48 hours. Slices were then cut into 150 *μ*m thick coronal sections with a vibratome, dehydrated with ascending alcohols, and mounted with epoxy resin between two coverslips.

To avoid any bias in the analysis, the slides were coded and the code was not broken until the analysis was completed. From each animal, six pyramidal neurons from layers III and V inside the mPFC were randomly selected. In order to be suitable for dendritic spine analysis, neurons should follow these features: (i) they must display complete Golgi impregnation of the apical dendrite, (ii) the cell type must be identifiable, and (iii) the minimum length of the apical dendrite must be 200 *μ*m from the soma. Each neuron was traced at 1000x magnification using a light microscope with a camera lucida drawing tube attachment (Nikon, Japan) and spines were quantified in four successive longitudinal segments of 50 *μ*m up to a total length of 200 *μ*m. Overall spine density values or densities per segment were expressed as the number of spines/*μ*m length. For each experimental group, mean ± SEM was determined and the resulting values were analyzed by one-way ANOVA with the number of animals as “*n*.” Significant effects were further analyzed by Bonferroni's post hoc test, using the IBM SPSS statistics software (version 19).

### 2.7. Immunohistochemistry for Confocal Microscopy

In order to study the peridendritic GABAergic innervation of mPFC pyramidal neurons after treatment, 50 *μ*m thick coronal sections were processed “free-floating” for immunohistochemistry as described above, but omitting the endogenous peroxidase block. Sections were incubated overnight with a cocktail of three primary antibodies: mouse IgG anti-CaMKII*α* (1 : 500, Abcam), mouse IgG anti-MAP2 (1 : 2000, Sigma-Aldrich), rabbit anti-GAD65/67 (1 : 1000, Chemicon-Millipore), and then with a cocktail of two secondary antibodies: donkey anti-mouse IgG-Alexa Fluor® 555 (1 : 400, Molecular Probes) and donkey anti-rabbit Alexa Fluor 488). Finally, sections were mounted on slides and coverslipped using DakoCytomation fluorescent mounting medium (Dako North America Inc., Carpinteria, CA). Both anti-CaMKII*α* antibody and anti-MAP2 primary antibody have been generated in mouse and were detected with the same anti-mouse fluorescent antibody in order to label together the soma and apical dendrite of pyramidal neurons.

### 2.8. Analysis of GAD65/67 Puncta Density throughout the Apical Dendrites of mPFC Pyramidal Neurons

Sections processed for fluorescent immunohistochemistry were observed under a confocal microscope (Leica TCS-SPE) using a 63x oil objective. Similar to the analysis of dendritic spine density using Golgi method, six pyramidal neurons from mPFC layers III and V were randomly selected from all the sections containing this region for each animal. These neurons were first identified using conventional fluorescence microscopy and then Z-series of optical sections (0.5 *μ*m apart) covering all its three-dimensional extension were acquired using sequential scanning mode. In order to be suitable for the study, neurons should have these features: (i) they had to express both CaMKII-*α* and MAP2, (ii) their soma shape had to be unequivocally pyramidal, and (iii) we should be able to follow clearly the apical dendrite up to 150 *μ*m from the soma. Stacks were processed with LSM 5 Image Browser software at 4x-Zoom magnification. The profile of these apical dendrites was drawn and puncta which are nonseparated by more than 0.5 *μ*m from the shaft were counted in four successive segments of 50 *μ*m up to a total length of 150 *μ*m. A puncta was defined as having an area not smaller than 0.15 and not larger than 2.5 *μ*m^2^ [13, 16]. The quantification of puncta was performed in several consecutive confocal planes covering the three-dimensional extension of each selected dendrite, in which the penetration of GAD65/67 and MAP2 antibodies was optimal. Overall puncta density values or densities per segment were expressed as the number of puncta/*μ*m of dendrite length. For each experimental group, mean ± SEM was determined and the resulting values were analyzed by one-way ANOVA with the number of animals as “*n*.” Significant effects were further analyzed by Bonferroni's post hoc test, using the IBM SPSS statistics software (version 19).

## 3. Results

### 3.1. PPHT Treatment Does Not Change VGLUT1 Expression in the mPFC Neuropil, but PSA Depletion Decreases It

**Figure 2:**
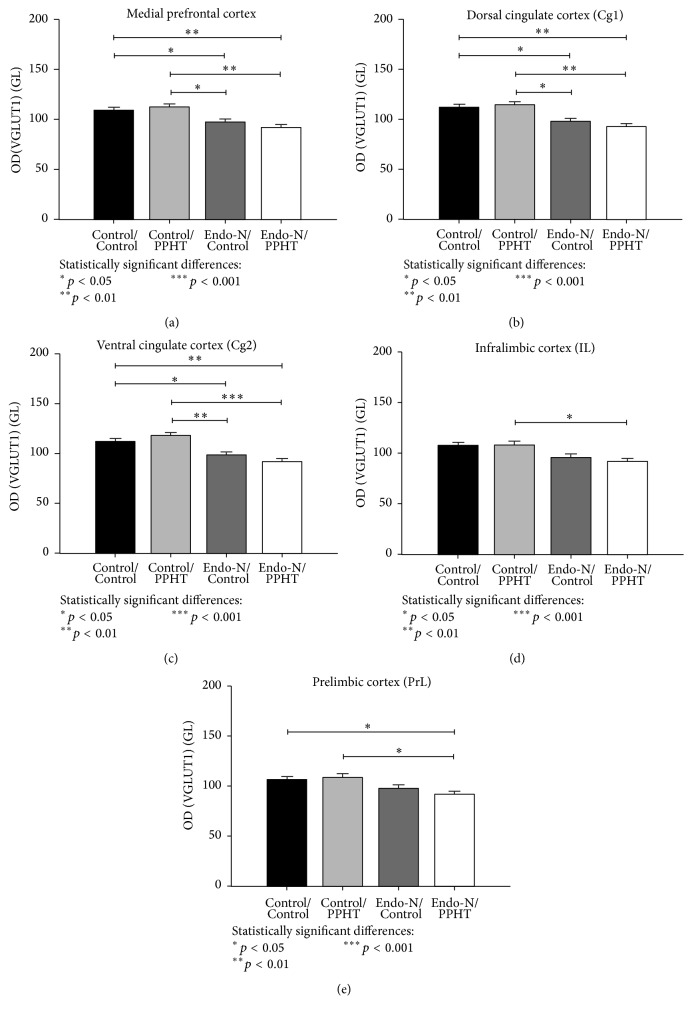
Graphs showing changes in the neuropil of VGLUT1 in the mPFC (a), dorsal cingulate (b), ventral cingulate (c), infralimbic (d), and prelimbic (e) cortices after Endo-N and/or PPHT treatments. Three sections per animal (*n* = 6 animals/group) were examined under bright-field illumination, homogeneously lighted, and digitalized using a CCD camera at 20x magnification. Grey levels were converted to optical densities (OD) using Image J software (NIH) (see Materials and Methods). Error bars represent SEM and asterisks in bars indicate statistically significant differences between groups after univariate repeated measures ANOVA followed by multiple pairwise comparisons with Bonferroni's correction (^*∗*^ *p* < 0.05,  ^*∗∗*^ *p* < 0.01, and ^*∗∗∗*^ *p* < 0.001).

For VGLUT1 neuropil expression (Figures 2(a)–2(e)), repeated measures ANOVA test showed significant main effects of* treatment* (*F*_3,18_ = 9.689, *p* < 0.001),* region* (*F*_3,16_ = 6.829, *p* = 0.004), and* layer* (*F*_4,15_ = 168.327, *p* < 0.001). Consequently, multiple pairwise comparisons with Bonferroni's correction were performed in order to compare treatment effect on the whole mPFC between pairs of experimental groups. Our results indicate that VGLUT1 expression in the mPFC neuropil was not affected by PPHT treatment, neither in the presence nor in the absence of PSA (Control/Control versus Control/PPHT or Endo-N/Control versus Endo-N/PPHT: *p* = 1.000), but PSA depletion by itself decreased it (Control/Control versus Endo-N/Control: *p* = 0.028; Control/Control versus Endo-N/PPHT: *p* = 0.004) (Figure 2(a)).

The two-way interaction “*region* ×* treatment*” on the repeated measures ANOVA test was significant for VGLUT1 neuropil expression (*F*_3,18_ = 6.703, *p* = 0.003). Consequently, multiple pairwise comparisons with Bonferroni's correction were performed in order to compare treatment effect between pairs of experimental groups on each region within the mPFC (Cg1, Cg2, IL, and PrL) (see *p* values on Figures 2(b)–2(e)). Results indicate that the same effects of those described above for VGLUT1 expression considering the whole mPFC neuropil (Figure 2(a)) can be observed in the dorsal cingulate cortex (Cg1) and ventral cingulate cortex (Cg2) (see *p* values on Figures 2(b) and 2(c)). However, in the infralimbic cortex (IL), there were only significant decreases in VGLUT1 expression when compared to Control/PPHT versus Endo-N/PPHT (see *p* values on Figure 2(d)). In the prelimbic cortex (PrL), significant decreases were observed when compared to Control/Control or Control/PPHT versus Endo-N/PPHT (see *p* values on Figure 2(e)).

**Figure 3:**
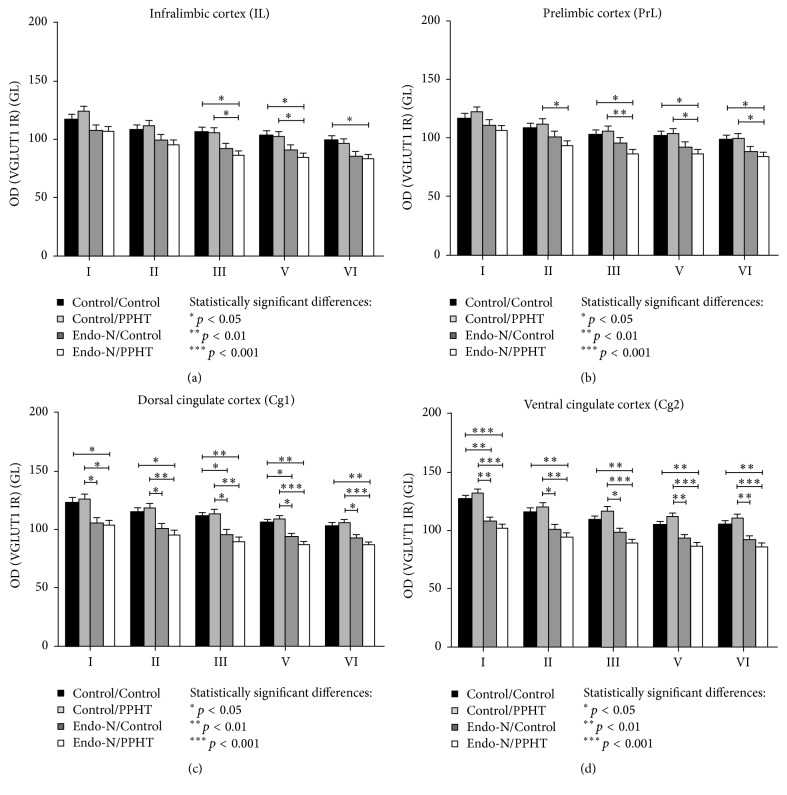
Graphs representing the changes in VGLUT1 neuropil expression after Endo-N and/or PPHT treatments in the different layers and regions within the mPFC. (a) Infralimbic cortex; (b) prelimbic cortex; (c) dorsal cingulate cortex; (d) ventral cingulate cortex. Six animals per group were analyzed. Error bars represent SEM and asterisks in bars indicate statistically significant differences between groups after univariate repeated measures ANOVA followed by multiple pairwise comparisons with Bonferroni's correction (^*∗*^ *p* < 0.05, ^*∗∗*^ *p* < 0.01, and ^*∗∗∗*^ *p* < 0.001). Roman numbers indicate cortical layers.

“Region × layer × treatment” interaction was also statistically significant (F_12,9_ = 3.484, *p* = 0.035) for VGLUT1 neuropil expression. Therefore, multiple pairwise comparison with Bonferroni's correction was performed in order to compare treatment effects among layers in every region within the mPFC. Results indicate that all layers within Cg1 and Cg2 cortices were affected by Endo-N treatment in combination with PPHT (Control/Control versus Endo-N/PPHT; see *p* values on Figures 3(c) and 3(d)), while, in the IL and PrL cortices, VGLUT1 expression was only reduced by this combined treatment in layers III, V, and VI (see *p* values on Figures 3(a) and 3(b)). By contrast, Endo-N treatment alone only decreased VGLUT1 expression in some layers of the cingulate cortex (Cg1, layers III and V; Cg2, layer I) (Control/Control versus Endo-N/Control; see *p* values on Figure 3).

### 3.2. PPHT and Endo-N by Themselves or in Combination Decrease Dendritic Spine Density in mPFC Pyramidal Neurons

**Figure 4:**
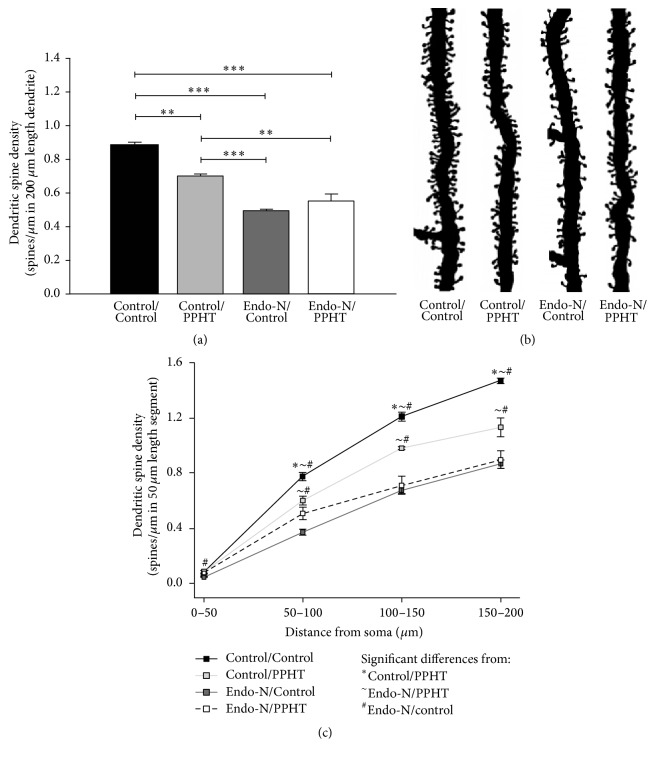
Dendritic spine density in mPFC pyramidal neurons after PPHT or Endo-N treatment and their combination. (a) Graph showing statistically significant differences between groups (*n* = 6 animals/group) when considering the total length (200 *μ*m) of the measured dendrites. (b) Camera lucida drawings of 50 *μ*m length dendritic segments located 150–200 *μ*m distal from the soma in dorsal cingulate cortex (Cg1), layer III. Note the decreased number of dendritic spines in all treated groups when compared with Control/Control group. (c) Graph representing dendritic spine density as a function of distance from the soma in 50 *μ*m length segments (*n* = 6 animals/group). Error bars represent SEM and symbols in bars indicate statistically significant differences between groups (see graph legend) after one-way ANOVA followed by Bonferroni's correction. For graph (a), ^*∗*^ *p* < 0.05, ^*∗∗*^ *p* < 0.01, and ^*∗∗∗*^ *p* < 0.001. For graph (c), please see *p* values in Results.

PPHT treatment induced a statistically significant overall decrease in spine density in the apical dendrites of pyramidal neurons (Control/Control versus Control/PPHT; *p* = 0.001; 20.47% decrease) (Figures 4(a) and 4(b)). Considering the distance from the soma, decreases in the spine density were only statistically significant from the second 50 *μ*m length segment onwards (1st segment: *p* = 1.000; 2nd segment: *p* = 0.012; 3rd segment: *p* = 0.008; 4th segment: *p* = 0.001) (Figure 4(c)).

PSA depletion by itself also produced an overall 44.3% decrease in spine density throughout the apical dendrites (200 *μ*m length) of mPFC pyramidal neurons (Control/Control versus Endo-N/Control: *p* < 0.001) (Figures 4(a) and 4(b)). Statistically significant decreases in spine density were also found in the four distal 50 *μ*m length segments in which the apical dendrites were divided (1st segment: *p* = 0.002; 2nd segment: *p* < 0.001; 3rd segment: *p* < 0.001; 4th segment: *p* < 0.001) (Figure 4(c)).

When PSA was removed before PPHT treatment, the decrease in dendritic spine density was higher than the decrease induced by PPHT treatment by itself (37.73%) (Control/Control versus Endo-N/PPHT: *p* < 0.001) (Figures 4(a) and 4(b)). Decreases were statistically significant from the second to the fourth 50 *μ*m length segment (1st segment: *p* = 1.000; 2nd segment: *p* < 0.001; 3rd segment: *p* < 0.001; 4th segment: *p* < 0.001) (Figure 4(c)).

No statistically significant changes in spine density were detected when Endo-N/Control group was compared with Endo-N/PPHT group (*p* = 0.769) (Figures 4(a) and 4(b)).

### 3.3. The Density of GAD65/67 Expressing Puncta Opposed to the Dendrites of mPFC Pyramidal Neurons Does Not Change after PPHT or Endo-N Treatments

**Figure 5:**
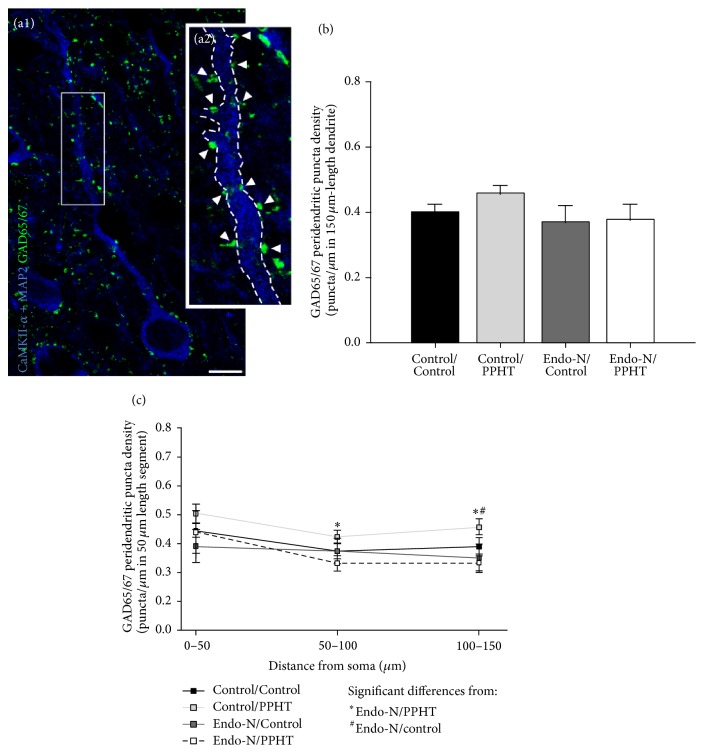
Confocal microscopic analysis of GAD65/67 expressing puncta in the peridendritic region of mPFC pyramidal neurons after Endo-N or PPHT treatments and their combination. (a1-a2) Single confocal planes showing a pyramidal neuron somata and its apical dendrite (immunolabeled for CaMKII-*α* and MAP2; blue color and marked with dashed line) innervated by GABAergic puncta (GAD65/67 immunoreactive puncta; green color and marked with arrowheads). (a2) is a 3x magnification of the squared section in (a1). Scale bar for (a1): 10 *μ*m. (b) Graph showing no changes in the peridendritic density of GAD65/67 expressing puncta (puncta/*μ*m) when considering the total length (200 *μ*m) of the measured dendrites. (c) Graph representing GAD65/67 peridendritic puncta density as a function of distance from the soma in 50 *μ*m length segments. In both graphs, 6 animals per group were analyzed. Error bars represent SEM and symbols in bars indicate statistically significant differences between groups after one-way ANOVA followed by Bonferroni's correction (see graph legend).

Contrary to what we described previously, using the same PPHT treatment for the inhibitory puncta surrounding the somata of mPFC pyramidal neurons [13], the density of GAD65/67 expressing puncta opposed to the first 150 *μ*m of their dendrites did not change after PPHT or Endo-N treatments, alone or in combination (Control/Control versus Control/PPHT: *p* = 0.214; Control/Control versus Endo-N/Control: *p* = 0.542; Control/Control versus Endo-N/PPHT: *p* = 0.618) (Figures 5(a) and 5(b)). Statistically significant decreases in the density of GAD65/67 expressing puncta were only observed between Control/PPHT and Endo-N/PPHT treated animals in the second and third 50 *μ*m length segments (*p* = 0.046 and *p* = 0.024, resp.) and between Control/PPHT and Endo-N/Control animals in the third 50 *μ*m length segment (*p* = 0.043) (Figure 4(c)).

## 4. Discussion

The present results describe the effects of the D2R agonist PPHT and the depletion of PSA from NCAM on the dendritic spine density of pyramidal neurons, the GABAergic innervation of these dendrites, and the expression of the excitatory synapse marker VGLUT1 in the mPFC of adult rats. These results complement our previously published data on the expression of PSA-NCAM and molecules related to inhibitory neurotransmission and they add further knowledge to our understanding of the cellular and molecular basis of neuronal plasticity in the adult brain. In the following paragraphs, we discuss the effects of these treatments in light of previous evidence, hypothesize on the mechanisms underlying this plasticity, and explore their implication on the etiopathology of psychiatric disorders, especially on schizophrenia.

### 4.1. Effects of D2R Agonist Treatment on mPFC Neuronal Circuitry

This is, to our knowledge, the first report describing the effects of a selective D2R agonist on pyramidal neuron spine density. However, there are previous evidences suggesting that manipulation of dopamine levels modulates spine density in this cortical region. Chronic treatments with drugs that promote dopaminergic neurotransmission (among other effects), such as cocaine, phencyclidine, and amphetamine, have rendered conflicting results. Although there is evidence, at least in primates, that long term amphetamine treatment decreases spine density in the mPFC [17], there are also reports describing increases in spine density by cocaine [18] and in the density of axospinous synapses by cocaine [19] and phencyclidine [20]. By contrast, dopamine depletion from the mPFC, genetic deletions of D2R, or decreases in dopamine levels observed in schizophrenia or after chronic stress are also associated with reductions in spine density in this area [6, 21]. These results may indicate that dopamine has an inverted U-shaped influence on mPFC pyramidal neuron structure, similar to its influence on the function of this cortical region [22]. These effects of PPHT on spine density may be directly mediated by D2R present in pyramidal neurons (these receptors can be localized in their spines) [23], but also indirectly, through D2R present in interneurons [24]. Previous reports showed that D2R agonists decrease AMPA receptor expression in mPFC pyramidal neurons [25], which in turn decreases spine density in these cells [26].

Since VGLUT1 expression is not affected in the mPFC neuropil, the number of excitatory synapses may not change after chronic PPHT treatment. This is apparently in contrast with the decrease in spine density detected in the apical dendrites of pyramidal neurons, since these postsynaptic structures are mainly contacted by excitatory inputs. However, it is possible that most synapses contacting the spines may not have disappeared but may persist, contacting the dendritic shaft. Our previous results showing that PPHT increases synaptophysin (SYN) expression in the neuropil [12, 13] are also apparently in contrast to this absence of change in VGLUT1 expression. However, it is likely that the increase in the number of synapses suggested by the increase in SYN expression was due to an increase in inhibitory synapses, as suggested by the increase in GAD67 expression found in our previous studies. Nevertheless, as the present results indicate, this increase in the number of inhibitory synapses does not appear to be located on the principal dendrite.

Taking together our previous results [12, 13] and the present ones, it appears that chronic PPHT treatment may induce an attenuation of excitatory neurotransmission in the mPFC, probably via an increase in inhibitory synapses, both in the neuropil and in the perisomatic region, but not in the apical dendrite, of pyramidal neurons. This is in consonance with the findings of a previous report, which described that the activation of D2R attenuated excitatory synaptic transmission in the adult PFC, probably involving GABA release by local interneurons [27].

### 4.2. Effects of PSA Depletion and Its Influence on D2R-Induced Changes in Prefrontocortical Circuitry

The present results demonstrate that PSA depletion from the mPFC neuropil downregulates the expression of VGLUT1, a marker of excitatory synapses, and that this effect is not perturbed by a subsequent PPHT treatment. Since in this cortical region PSA-NCAM expression is restricted to interneurons, the effect of the depletion of this complex sugar on principal neurons should be indirect. It is possible that the excitatory synapses affected by this depletion were those contacting interneurons, which previously expressed PSA-NCAM. Alternatively, the effect of PSA depletion from these interneurons may result in changes in interneuronal structure or connectivity that finally influence indirectly excitatory terminals. Although Endo-N treatment blocks the effect of PPHT on GAD67 expression in the neuropil, it has no effect when administered alone [13]. Consequently, PSA depletion by itself does not seem to have a general effect (neuropil) on inhibitory neurotransmission in the mPFC. In agreement with these results, in the present study, we could not find any changes in the density of GAD65/67 expressing puncta on the principal dendrites of pyramidal neurons after Endo-N treatment alone or in combination with PPHT. However, as we have previously demonstrated that PSA depletion has an important impact on the perisomatic inhibition of pyramidal neurons in this region (increasing the number of GAD65/67 and PV expressing puncta and the proportion of PV puncta coexpressing SYN), the lack of effect on peridendritic inhibition was a bit unexpected [13]. A possible explanation for these effects is that the removal of PSA from NCAM may activate some synapses only from PV expressing interneurons (perisomatic synapses), which were previously blocked by the presence of PSA. Other classes of interneurons targeting the dendrites of pyramidal cells (double-bouquet, Martinotti, neurogliaform cells) are probablynot affected by this treatment. Consequently, the expression of molecules related to active inhibitory neurotransmission, such as GAD65/67, is increased and more puncta become detectable in the perisomatic but not in the peridendritic region of pyramidal neurons [13]. These results are in agreement with a previous study on the developing visual cortex, in which PSA depletion induced precocious maturation of perisomatic innervation by basket interneurons, resulting in enhanced inhibitory synaptic transmission [16]. Thus, the presence of PSA-NCAM in control adult mPFC may act as a regulator of perisomatic inhibitory innervation. Consequently, the removal of PSA may increase the inhibition of pyramidal neurons, probably affecting specifically the perisomatic puncta arising from basket neurons. This increase in perisomatic inhibition may well be responsible for the decrease in VGLUT1 expression in the mPFC neuropil. A previous study did not find significant differences in VGLUT1 expression after chronic treatment with diazepam, a GABAA receptor agonist, although a tendency for an increase was observed in the cerebral cortex [28].

PSA removal from the mPFC also induces dramatic effects on dendritic spine density in pyramidal neurons. Since these excitatory cells do not express PSA-NCAM during adulthood [11], this loss of spines must be a secondary effect mediated by interneurons. In fact, the level of decrease in dendritic spine density was comparable between Endo-N treated animals and animals treated with Endo-N and PPHT. As we have discussed above for the changes in VGLUT1 expression, the decrease in spine density found in mPFC pyramidal neurons after Endo-N treatment may be induced by the putative enhancement of perisomatic inhibition suggested by the increase in the number of GAD65/67 and PV expressing puncta in this region. Unfortunately, we have failed to find published data on the effects of enhanced inhibition on the dendritic structure of cortical pyramidal neurons. It is interesting to note, however, that a reduction in the density of dendritic spines and a concomitant elevation of GABAergic signaling has been found recently in cortical pyramidal neurons of mice treated with an estrogen receptor ligand [29]. Moreover, reduced NMDA receptor function and expression in pyramidal neurons have been found after chronic treatment with benzodiazepines [30]. It is possible that these alterations in NMDA receptors induced by an increased inhibition, similar to that suggested after PSA depletion, influence the decrease in spine density. In fact, treatments with NMDA receptor antagonists decrease the number of asymmetric spine synapses [20] and spine density [31] on mPFC pyramidal neurons; and loss of GluN2B-containing NMDA receptors in the cortex also reduces dendritic spine density [32]. Moreover, the effects of estrogens on pyramidal neuron spine density are mediated by NMDA receptors [33].

The changes induced by PSA depletion or by alterations in the degree of NCAM polysialylation may be caused not only by the regulation of NCAM adhesive properties, but also by the influence of PSA on NCAM-mediated signaling (see [8] for review). Moreover, interference on NCAM may also impair dopaminergic neurotransmission, since NCAM regulates the trafficking, internalization, and degradation of D2R [34]. These alterations in dopaminergic neurotransmission induced by the interference on NCAM signaling may also explain why the depletion of PSA from the mPFC has effects on spine density similar to those observed after PPHT treatment.

### 4.3. Implications of the Present Findings in Psychiatric Disorders

The present results may have important implications for our understanding of the neurobiological basis of different psychiatric disorders, especially schizophrenia. It is known that dopamine and D2R are important players on the etiopathogenesis and the therapeutics of this disorder [35] and that excessive D2R activation might contribute to prefrontal dysfunction in schizophrenia [1, 2]. The decrease in pyramidal spine density is a common finding in the PFC of schizophrenic patients [36], in animal models of this psychiatric disorder [5, 37, 38], and after experimental manipulation of mPFC dopamine levels [6]. There is also evidence for a relationship between dysregulation of NCAM and its posttranslational modifications and the neural abnormalities found in different mood disorders [39]. Moreover, one of the enzymes responsible for NCAM polysialylation, St8SiaII, is a candidate susceptibility gene for schizophrenia [40]; and alterations in PSA-NCAM expression have been found in schizophrenic patients and animal models [41–43]. Our results showing the involvement of PSA-NCAM in the plasticity induced by D2R manipulation, its necessary presence for the regulation of mPFC perisomatic inhibition, and its effects on dendritic spine density also support the idea that altered PSA-NCAM expression may participate in the pathogenesis of this psychiatric disorder.
